# Taxonomic assessment of phlebotomine sand flies in Southeast Asia based on records from Cambodia

**DOI:** 10.1016/j.crpvbd.2026.100356

**Published:** 2026-01-29

**Authors:** Didot Budi Prasetyo, Thibault Vallecillo, Eva Krupa, Julian Gratiaux, Alicia Vol, Mathieu Loyer, Jean-Philippe Martinet, Quentin Di Brasi, Khamsing Vongphayloth, Bruno Mathieu, Sébastien Boyer, Arezki Izri, Frédérick Gay, Jérôme Depaquit

**Affiliations:** a UR ESCAPE, Université de Reims Champagne-Ardenne, USC ANSES PETARD, Reims, 51100, France; b Medical and Veterinary Entomology Unit, Institut Pasteur du Cambodge, Phnom Penh, Cambodia; c Centre Hospitalo-Universitaire, Pharmacie centrale, Reims, 51100, France; d Institut Pasteur, Université Paris Cité, CNRS UMR 2000, INRAE USC 1510, Ecology and Emergence of Arthropod-borne Pathogens Unit, Paris, 75015, France; e Institut Jean Godinot, Reims, France; f Institut Pasteur du Laos, Laboratory of Vector-Borne Diseases, Samsenhai Road, Ban Kao-Gnot, Sisattanak District, Vientiane, 3560, Laos; g Institut de Bactériologie et Parasitologie, UR3073 PHAVI, Université de Strasbourg, 3 rue Koeberlé, Strasbourg, France; h Parasitologie-Mycologie, CHU Avicenne, Université Paris 13, Bobigny, UMR 190, Unité des Virus émergents, Marseille, France; i Sorbonne Université, INSERM, Institut Pierre Louis d’Epidémiologie et de Santé Publique, Hôpital Pitié-Salpêtrière, APHP, Paris, 75013, France; j Centre Hospitalo-Universitaire, Pôle de Biologie Territoriale, Laboratoire de Parasitologie-Mycologie, Reims, 51100, France

**Keywords:** Phlebotomine sand flies, Cambodia, Southeast Asia, Taxonomy, DNA barcoding, Molecular systematics

## Abstract

Previously considered a leishmaniasis-free region, Southeast Asia has reported emerging cases over the past 20 years. This has renewed interest in the primary vectors, the phlebotomine sand flies. However, information on these vectors in Southeast Asia, particularly in Cambodia, remains scarce. To update distribution records and assess species diversity, CDC light traps were deployed to collect sand flies at 16 locations across several provinces of Cambodia in 2011 and 2014. A total of 940 sand flies were collected and identified both morphologically and molecularly using cytochrome *b* as a marker gene. Species identification revealed the presence of four genera and 10 species. The predominant species was *Grassomyia* sp. (21.9%), followed by *Sergentomyia sylvatica* (21.4%) and *Sergentomyia iyengari* Group (16.7%). Generally identified as *Grassomyia indica*, the *Grassomyia* specimens found in this study exhibited morphological disparities, although genetic analysis showed homogeneity with *Gr. indica* from other countries. *Sergentomyia iyengari* and *Sergentomyia barraudi* each displayed two distinct groups based on both morphological and genetic analyses, suggesting that different species within these complexes are present in Cambodia. Further investigation of these species complexes is warranted, since genomic evidence suggests the existence of previously undescribed species in this region.

## Highlights

• First inventory of phlebotomine sand flies in Cambodia.
• Record of 10 species.
• Discussion of the *Sergentomyia barraudi* and *Se. iyengari* species complexes.
• *Phlebotomus kiangsuensis* and *Sergentomyia anodontis* are species complexes.

## Graphical abstract

**Figure:**
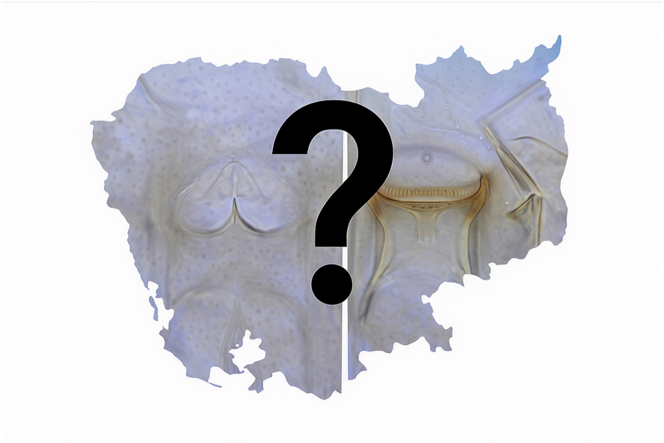

## 1 Introduction

Phlebotomine sand flies (Diptera: Psychodidae) are medically important vectors of several pathogens, most notably *Leishmania* parasites, which cause leishmaniases in humans and animals (Maroli et al., 2013). In Southeast Asia, the role of sand flies in disease transmission has historically received limited attention compared to other vectors such as mosquitoes transmitting the agents of malaria and dengue (Hustedt et al., 2022).

Historically, Southeast Asia was considered free of leishmaniasis. The first autochthonous cases occurred in Vietnam in 2002, although they remained confidential (Taverne, 2002). Since 2007, however, Thailand has become a recognised focus of locally transmitted leishmaniasis (Bualert et al., 2012; Leelayoova et al., 2017). To date, three species, i.e. *Leishmania martiniquensis*, *Leishmania orientalis*, and *Leishmania donovani* complex, have been identified as causative agents of indigenous visceral leishmaniasis among Thai patients (Charoensakulchai et al., 2020). Despite these developments, there remains a lack of information related to phlebotomine sand flies from this part of the world. The most recent publications related to phlebotomine sand flies from Southeast Asia are almost exclusively from Thailand (Apiwathnasorn et al., 1989, 1993, 2011; Depaquit et al., 2006, 2009, 2019; Muller et al., 2007; Polseela et al., 2007, 2011a, 2011b, 2016; Curler, 2011; Sukra et al., 2012; Kanjanopas et al., 2013; Panthawong et al., 2015; Phumee et al., 2017, 2024; Sor-Suwan et al., 2017; Jaturas et al., 2018; Thammapalo et al., 2020; Phuphisut et al., 2021; Somwang et al., 2021; Jariyapan et al., 2022; Preativatanyou et al., 2023; Renaux Torres et al., 2023; Sunantaraporn et al., 2024).

Within this context, Cambodia remains relatively underexplored in terms of sand fly fauna. A bibliographic search carried out on PubMed on 3rd July 2025, with the words “Cambodia” and “*Phlebotomus*” or “*Sergentomyia*” returned only two results, excluding affiliations matching. One is a paper describing a new species of *Idiophlebotomus* from the country (Loyer et al., 2016), and the other is a review paper focusing on phlebotomine sand flies of the Greater Mekong subregion (Hustedt et al., 2022). The majority of the records are old, except for the recent inclusion of the species examined in the present study in an update of the sand flies of the world (Galati et al., 2025). Most records date back to the 1930s, when Cambodia was under colonial administration, and precede the Khmer Rouge period, which does not help with the search for bibliographic sources (Raynal, 1935; Raynal and Gaschen, 1935a, 1935b, 1935d). Three more recent articles published between the 1960s and 1980s included some records from Cambodia in review articles (Quate, 1962; Lewis, 1978, 1987).

This study investigates the taxonomy of phlebotomine sand flies in Cambodia through an integrated approach combining morphological observation and cytochrome *b* sequence analyses, providing new insights into species composition and the evolutionary relationships of local sand fly populations.

## 2 Materials and methods

### 2.1 Sand fly collection

**Fig. 1:**
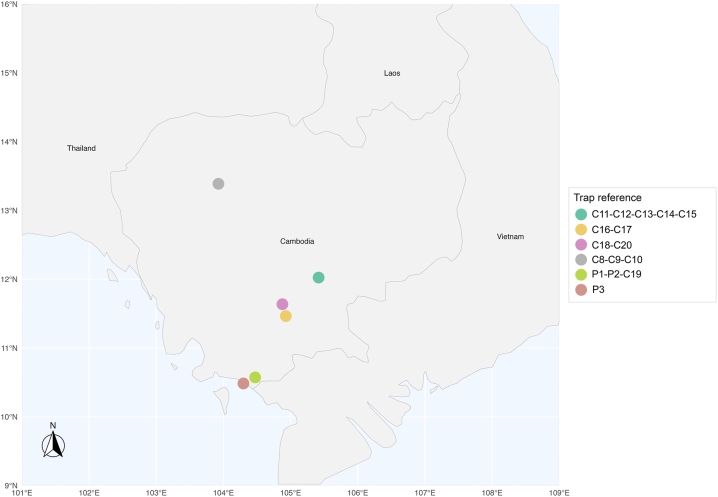
Trapping locations.

Two habitat types in different parts of the country (Fig. 1) were selected as collection sites: (i) caves and cave entrances; and (ii) rural environment near domestic animals. During two collection periods (2011 and 2014), phlebotomine sand flies were collected overnight (from 17:00 to 7:00 h) using standard CDC light traps (John W. Hock Company, Gainesville, FL, USA) deployed at multiple sampling points (Table 1). Collected specimens were sorted and preserved in 95% ethanol for subsequent analyses.

**Table 1:** Trapping locations.

Date of capture (DMY)	Trap	Location	Biotope	Climate	GPS coordinates	No. of CDC traps
25.12.2011	P1	Kampong Trach Mountain, Kampot Province	In cave	26 °C, 70% RH, no wind	10°34′23.7"N, 104°28′20.8"E	2
25.12.2011	P2	Kampong Trach Mountain, Kampot Province	Outside cave	26 °C, 81% RH, no wind	10°34′23.8"N, 104°28′12.3"E	1
26.12.2011	P3	Kep National Parc	Outside cave	26 °C, 81% RH, no wind	10°29′07.9"N, 104°17′49.2"E	3
27.04.2014	C8	Kok Chan, Siem Reap Province	Cow and chicken shelters	29 °C, 78% RH, 1 km/h wind	13°23′20"N, 103°55′30"E	3
27.04.2014	C9	Kok Chan, Siem Reap Province	Cow shelters	29 °C, 78% RH, 1 km/h wind	13°23′04"N, 103°55′26"E	2
28.04.2014	C10	Prey Koy (Rolous Chas), Siem Reap Province	Goat shelters	29 °C, 78% RH, 1 km/h wind	13°22′36"N, 103°55′20"E	4
29.04.2014	C11	Roka Knong, Kampong Cham Province	Cow, pig and chicken shelters	29 °C, 78% RH, 3 km/h wind, heavy rainfall and storm overnight	11°56′50"N, 105°23′45"E	5
29.04.2014	C12	Roka Knong, Kampong Cham Province	Cow, pig and chicken shelters	29 °C, 78% RH, 3 km/h wind, heavy rainfall and storm overnight	11°56′50"N, 105°23′45"E	2
30.04.2014	C13	Roka Leu, Kampong Cham Province	Goat shelters	26 °C, 89% RH, no wind	11°58′34"N, 105°27′27"E	2
30.04.2014	C14	Roka Knong, Kampong Cham Province	Cow shelters	26 °C, 89% RH, no wind	11°56′50"N, 105°23′45"E	4
30.04.2014	C15	Andong Cros, Kampong Cham Province	Goat shelters	26 °C, 89% RH, no wind	12°1′29"N, 105°25′0"E	4
1.05.2014	C16	Prek Ho Lech, Takmao, Phnom Penh	Cow shelters	26 °C, 85% RH, 1 km/h wind	11°27′55"N, 104°55′46"E	8
1.05.2014	C17	Prek Ho Lech, Takmao, Phnom Penh	Rabbit shelters	26 °C, 85% RH, 1 km/h wind	11°27′15"N, 104°55′50"E	1
1.05.2014	C18	Svay Pak, Russey Keo, Phnom Penh	Goat shelters	26 °C, 85% RH, 1 km/h wind	11°37′56"N, 104°52′48"E	2
2.05.2014	C19	Angkor Chey, Kampot Province	In caves	34 °C, 49% RH, no wind	10°33′51"N, 104°29′05"E	6
3.05.2014	C20	Svay Pak, Russey Keo, Phnom Penh	Goat shelters	26 °C, 85% RH, 1 km/h wind	11°38′17"N, 104°52′43"E	4

*Abbreviation*s: DMY, day-month-year format; RH, relative humidity.

### 2.2 Sample processing and morphological analysis

Specimens dedicated to morphological analysis were mounted *in toto*: whole specimens were processed in batches. Soft tissues were lysed overnight in 10% KOH, rinsed, bleached in Marc-André solution (Abonnenc, 1972), rinsed again, and then dehydrated in successive ethanol baths (70%, 96%, absolute ethanol) and mounted between a microscope slide and cover slip in Euparal® mounting medium.

Specimens dedicated to molecular studies were partially mounted: wings, head and genitalia were cut off in a drop of absolute ethanol. Wings were directly mounted in Euparal®. Thorax and body were transferred to a 1.5-ml vial and stored at −20 °C until further analysis. Head and genitalia were processed as described above.

The mounting slides were examined using an Olympus BX50 microscope equipped with a DP26 camera. Voucher material was deposited in the Cambodian Collection of Phlebotomine Sand Flies at the Department of Parasitology of the Faculty of Pharmacy, University of Reims Champagne-Ardenne, and are available on request.

### 2.3 Molecular analysis

Genomic DNA was extracted from the first abdominal segments of individual sand flies using the QIAmp DNA Mini Kit® (Qiagen, Hilden, Germany) following the manufacturer’s instructions with minor modifications: sand fly tissues were homogenised using a pellet pestle (Treff, Switzerland), and DNA was eluted in 200 μl.

A fragment of cytochrome *b* (*cyt b*) gene was amplified by conventional PCR according to previously published conditions (Esseghir et al., 1997) using the primers C3B-PDR: 5′-CAY ATT CAA CCW GAA TGA TA-3′ and N1N-PDR: 5′-GGT AYW TTG CCT CGA WTT CGW TAT GA-3’. Subsequent sequencing reactions were performed using the same primers.

Although this study does not aim to perform a full phylogenetic analysis, it uses cytochrome *b* sequences to support taxonomic identifications. A Neighbour-Joining (NJ) tree was constructed in MEGA 11 (Tamura et al., 2021) using aligned sequences generated from this study together with reference sequences retrieved from GenBank. A sequence of the species *Chinius eunicegalatiae* (GenBank: PQ151807) was selected to serve as the outgroup. Estimation of evolutionary divergence was analysed with MEGA 11 using pairwise genetic distances (uncorrected p-distance), expressed as the number of base substitutions per site, with standard error estimates obtained through bootstrap resampling (100 replicates).

### 2.4 Mapping

Maps were created with RStudio 2023.12.0 using public domain data from Natural Earth with the packages *rnaturalearth*, *rnaturalearthdata*, *ggplot2*, *ggspatial*, *dplyr*, *readxl* and *RColorbrewer* (Wickham, 2016; Neuwirth, 2022; Dunnington, 2023; Massicotte and South, 2023; Pebesma and Bivand, 2023; R Core Team, 2023; Wickham et al., 2023; South et al., 2024; Wickham and Bryan, 2025).

## 3 Results

### 3.1 Morphological analysis

A total of 940 phlebotomine sand flies were collected and processed, representing 4 genera and 10 species: 5 *Idiophlebotomus* spp.; 80 *Phlebotomus* spp.; 649 *Sergentomyia* spp.; and 206 *Grassomyia* spp. (Table 2). Due to damaged specimens, we were not able to identify 24 *Sergentomyia* at the species level.

**Table 2:** Species inventory.

Trap ID	*Id. nicolegerae*		*Ph. stantoni*		*Ph. kiangsuensis*		*Se. iyengari* Group		*Se. barraudi* Group		*Se. hivernus*		*Se. bailyi*		*Se. sylvatica*		*Se. anodontis*		*Grassomyia* sp.		*Sergentomyia* sp.		Total		
	♀	**♂**	♀	**♂**	♀	**♂**	♀	**♂**	♀	**♂**	♀	**♂**	♀	**♂**	♀	**♂**	♀	**♂**	♀	**♂**	♀	**♂**	♀	**♂**	**♀+♂**
P1		4	1	2	39	19	1	1	1						35	25	8	12	1	3			86	66	152
P2	1		1	1	4	4	2	1	3	2	1				55	25	21	12	10	16	1		99	61	160
P3							28			2	2											6	30	8	38
C8									2	4			1	1					1	2			4	7	11
C9			1						2	2									2	2			5	4	9
C10							6	9	23	10			3	7					8	18		4	40	48	88
C11							3	8	3	4									8	23		1	14	36	50
C12							2		6				4	3					5		5	1	22	4	26
C13							2		1														3	0	3
C14				1			35	12	12	1			17	6	1				31	14	1		97	34	131
C15			2	1			26	10	11	2	1		17	6	3				33	12			93	31	124
C16													1										1	0	1
C17																							0	0	0
C18							2	1	1	2			8	6	1				9		1		22	9	31
C19			1		1		2	5	2	1					29	27	1		1	1			37	34	71
C20			1					1	1				16	8					5	1			23	10	33
Total	1	4	7	5	44	23	109	48	68	30	4	0	67	37	124	77	30	24	114	92	8	12	576	352	928

### 3.2 Molecular analysis

**Fig. 2:**
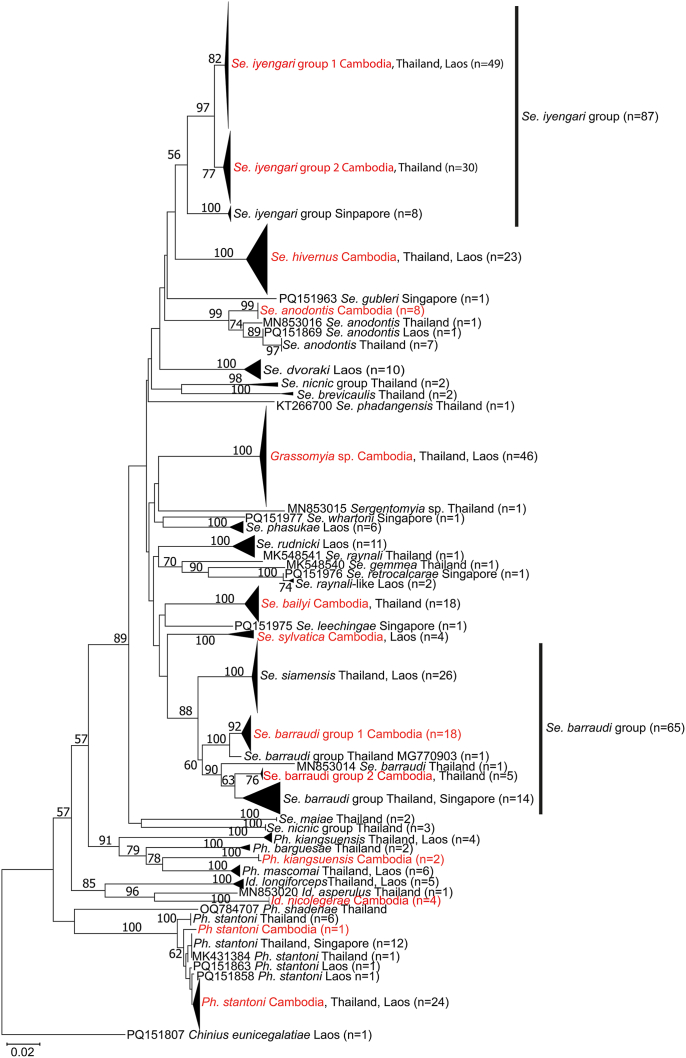
Neighbour-Joining tree obtained from a dataset of 439 cytochrome *b* sequences including 84 newly generated sequences from Cambodia. There was a total of 562 positions in the final dataset, including 303 variable positions, of which 258 are parsimony informative. The percentage of replicate trees in which the associated taxa clustered together in the bootstrap test (100 replicates) is shown next to the branches if > 50%. The *Se. iyengari* Group excludes the valid and well-differentiated species *Se. hivernus* and *Se. gemmea*. *Chinius eunicegalatiae* was used to root the tree.

Excluding the sequences of *Idiophlebotomus nicolegerae* previously deposited in GenBank (Loyer et al., 2016), we provide and analyze 84 new sequences deposited under the accession numbers PX864485-PX864568: 11 *Se. iyengari* Group (6 from the population *Se. iyengari* Group 1 and 5 from the population Se. *iyengari* Group 2); 22 *Se. barraudi* Group (18 from the population *Se. barraudi* Group 1 and 4 from the population *Se. barraudi* Group 2); 8 *Se. anodontis*; 3 *Se. sylvatica*; 1 *Se. hivernus*; 17 *Se. bailyi*; 18 *Grassomyia* sp.; 2 *Ph. stantoni*; and 2 *Ph. kiangsuensis*. We compared these sequences to 294 sequences previously deposited in GenBank (Supplementary Table S1). A Neighbour-Joining tree based on the sequences dataset is shown in Fig. 2. Estimates of evolutionary divergence of these sand fly species are shown in Table 3, Table 4, Table 5, Table 6.

**Table 3:** Estimates of evolutionary divergence over sequence pairs between (regular) and within groups (bold) based on 65 *Phlebotomus* spp. nucleotide sequences.

	Species	1	2	3	4	5	6
1	*Ph. stantoni*	**0.010**					
2	*Ph. shadenae*	0.153	**NA**				
3	*Ph. barguesae*	0.215	0.183	**0.008**			
4	*Ph. mascomai*	0.173	0.157	0.117	**0.007**		
5	*Ph. kiangsuensis* Thailand, Laos	0.229	0.209	0.186	0.163	**0.000**	
6	*Ph. kiangsuensis* Cambodia	0.216	0.189	0.137	0.096	0.158	**0.000**

*Note*: NA (not applicable) denotes cases in which it was not possible to estimate evolutionary distances (group including only one sequence).

**Table 4:** Estimates of evolutionary divergence over sequence pairs between (regular) and within groups (bold) based on 79 nucleotide sequences of specimens belonging to the *Sergentomyia barraudi* Group.

	Species	1	2	3	4	5	6
1	*Se. barraudi* Group Thailand	**0**					
2	*Se. barraudi* Group 2 Cambodia, Thailand	0.0700	**0.001**				
3	*Se. siamensis* Thailand Laos	0.0689	0.0849	**0.005**			
4	*Se. barraudi* Group Singapore, Thailand	0.0722	0.0278	0.0890	**0.0160**		
5	*Se. barraudi* Thailand MN853014	0.0759	0.0789	0.0906	0.0706	**NA**	
6	*Se. barraudi* Group 1 Cambodia	0.0178	0.0684	0.0769	0.0701	0.0792	**0.0020**

*Note*: NA (not applicable) denotes cases in which it was not possible to estimate evolutionary distances (group including only one sequence).

**Table 5:** Estimates of evolutionary divergence over sequence pairs between (regular) and within groups (bold) based on 155 nucleotide sequences of specimens belonging to the *Sergentomyia iyengari* Group.

	Species	1	2	3	4	5	6	7	8	9
1	*Se. hivernus*	**0.011**								
2	*Se. iyengari* Group Singapore	0.082	**0.002**							
3	*Se. iyengari* Group 1	0.097	0.060	**0.005**						
4	*Se. iyengari* Group 2	0.087	0.057	0.029	**0.006**					
5	*Se. dvoraki*	0.116	0.085	0.111	0.104	**0.009**				
6	*Se. gemmea*	0.157	0.135	0.136	0.141	0.145	**NA**			
7	*Se. raynali-*like	0.144	0.139	0.151	0.146	0.136	0.096	**0.000**		
8	*Se. raynali*	0.137	0.121	0.129	0.127	0.144	0.107	0.119	**NA**	
9	*Se. retrocalcarae*	0.137	0.131	0.142	0.139	0.131	0.091	0.003	0.114	**NA**

*Note*: NA (not applicable) denotes cases in which it was not possible to estimate evolutionary distances (group including only one sequence).

**Table 6:** Estimates of evolutionary divergence over sequence pairs between (regular) and within groups (bold) based on 20 nucleotide sequences of specimens identified as *Sergentomyia anodontis*.

	Species	1	2	3	4
1	*Se. anodontis* Laos	**NA**			
2	*Se. anodontis* Thailand	0.008	**0.000**		
3	*Se. anodontis* Thailand MN853016	0.028	0.026	**NA**	
4	*Se. anodontis* Cambodia	0.030	0.041	0.045	**0.000**

*Note*: NA (not applicable) denotes cases in which it was not possible to estimate evolutionary distances (group including only one sequence).

## 4 Discussion

### 4.1 Current knowledge of sand fly diversity in Cambodia

The sand fly fauna of Cambodia recorded in the scientific literature includes 13 species belonging to four genera,i.e. *Idiophlebotomus nicolegerae*, *Phlebotomus* (*Anaphlebotomus*) *stantoni*, *Ph*. (*Euphlebotomus*) *kiangsuensis*, *Sergentomyia* (*Neophlebotomus*) *whartoni*, *Se*. (*Parrotomyia*) *barraudi*, *Se*. (*Par*) *kwangsiensis*, *Se*. (*Rondanomyia*) *iyengari* (*s.l.*), *Se*. (*Ron.*) *khawi*, *Se*. (*Ron.*) *perturbans*, *Se*. (*Ron.*) *sylvatica*, *Se*. *anodontis*, *Se. bailyi*, and *Grassomyia indica*. The aim of this article is to characterise the sand fly fauna of Cambodia and to critically evaluate species identifications proposed in this study and in previous regional surveys.

Species diversity is much greater in two bordering countries, Thailand and Laos, where 37 and 30 species have been reported to date (Apiwathnasorn et al., 1989, 1993, 2011; Depaquit et al., 2006, 2009, 2019; Muller et al., 2007; Leger et al., 2010; Curler, 2011; Polseela et al., 2011b, 2016; Sukra et al., 2012; Kanjanopas et al., 2013; Panthawong et al., 2015; Phumee et al., 2017, 2021a, 2021b, 2024; Sor-Suwan et al., 2017; Jaturas et al., 2018; Srisuton et al., 2019; Thammapalo et al., 2020; Phuphisut et al., 2021; Hustedt et al., 2022; Preativatanyou et al., 2023; Renaux Torres et al., 2023; Vongphayloth et al., 2023, 2024; Sunantaraporn et al., 2024; Galati et al., 2025; Klaiklueng et al., 2025; Soomro et al., 2025). Studies conducted in these countries are much more numerous, explaining this abundance. The virtual absence of studies conducted in Cambodia alone explains the limited knowledge of sand fly biodiversity.

Generally, entomological studies conducted in Southeast Asia record many species and often reveal new ones for science. In neighbouring Singapore, a recent study that revealed the presence of sand flies in that country for the first time has led to the description of four species new to science (Ding et al., 2025). Therefore, a genuine nationwide sand fly trapping policy would likely result in the identification of roughly the same number of species in Cambodia as in Thailand and Laos and possibly the description of new species.

### 4.2 Genus *Phlebotomus*

**Fig. 3:**
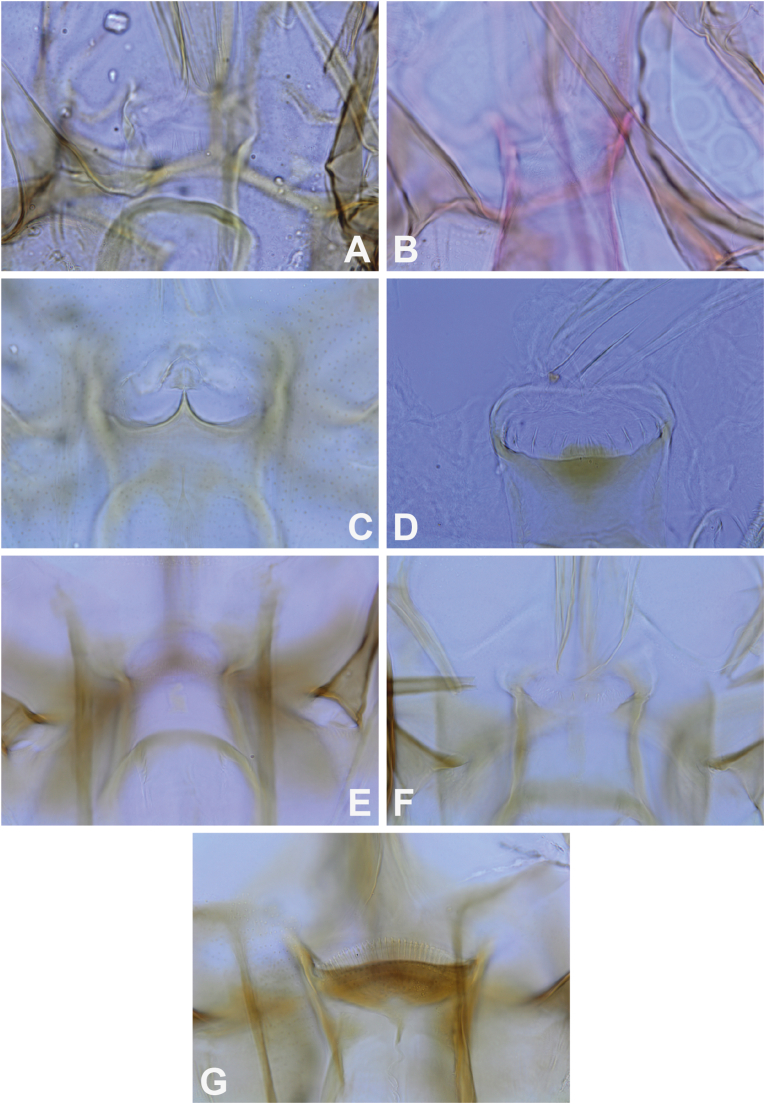
Cibarium in female *Ph. stantoni* labelled CAMB37 (**A**), *Ph. kiangsuensis*-like labelled CAMB487 (**B**), *Se. anodontis* labelled CAMB9 (**C**), *Se. hivernus* labelled CAMB211 (**D**), *Se. bailyi* labelled CAMB14 (**E**), *Se. sylvatica* labelled CAMB C19Ai03-79 (**F**), and *Grassomyia* sp. labelled CAMB141 (**G**).

Among the species captured during this study, two species of the genus *Phlebotomus* were found: *Ph. stantoni* and a species that was provisionally called *Ph. kiangsuensis*. Specimens of *Ph. stantoni* from Cambodia are morphologically comparable to those examined from Thailand, Laos, Vietnam, and Singapore. The cibarium of females generally displays four large, very sharp, translucent teeth (Fig. 3A). Molecular analysis indicates that all *Ph. stantoni* from Southeast Asia are grouped in a single clade (Fig. 2), with low intraspecific divergence (0.01; Table 3). Specimens of this species, which is widespread in Southeast Asia, were captured both in caves and in open environments.

In contrast, the taxonomy of *Ph. kiangsuensis* is more complex. This species was described from northern China in 1938 (Yao and Wu, 1938) and considered cave-associated. In Cambodia, it was detected exclusively in traps placed in caves or cave entrances (Table 2). According to the original description, the female has a pharynx that gradually enlarges posteriorly. The pharyngeal armature consists of a series of transverse ridges with 5 or 6 rows of teeth pointing backwards in the anterior middle of the armature (Fig. 3B). The spermathecae are sausage-shaped, with small transverse striations. The number of segments is not distinct but only shows faint transverse striations. At their base, a few crenulations show more clearly.

**Fig. 4:**
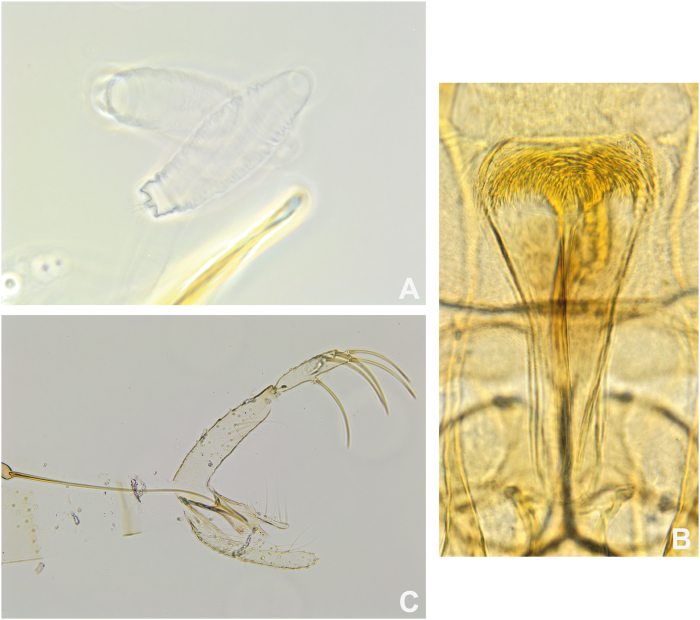
*Phlebotomus kiangsuensis* from Cambodia. **A** Spermathecae. **B** Pharynx in the female. **C** Male genitalia.

Males are described as being similar to those of *Ph. argentipes* but are distinguished by a larger middle lobe of the paramere than the other two ones and by the absence of the accessory spines of the parameral sheath. The examined specimens are generally consistent with the original description, notably with a distal ring of the spermathecae, in the shape of a brick placed on the body of the spermathecae (Fig. 4). However, the spermathecae of the specimens from Cambodia is striated whereas the drawing by Yao and Wu shows a rather smooth spermathecae (Yao and Wu, 1938). These differences may be attributable to improved visualisation technique (for example phase-contrast microscopy) unavailable at the time of original description. In addition, the pharynx of the specimens from Cambodia seems to be more armed and wider (Fig. 4) than that of the Chinese specimens used for the original description. Pending examination of type-material or topotypes, we refrain from assigning species-level status to this population.

### 4.3 Genus *Sergentomyia*: Taxonomic confusion in the *Se. iyengari* Group

**Fig. 5:**
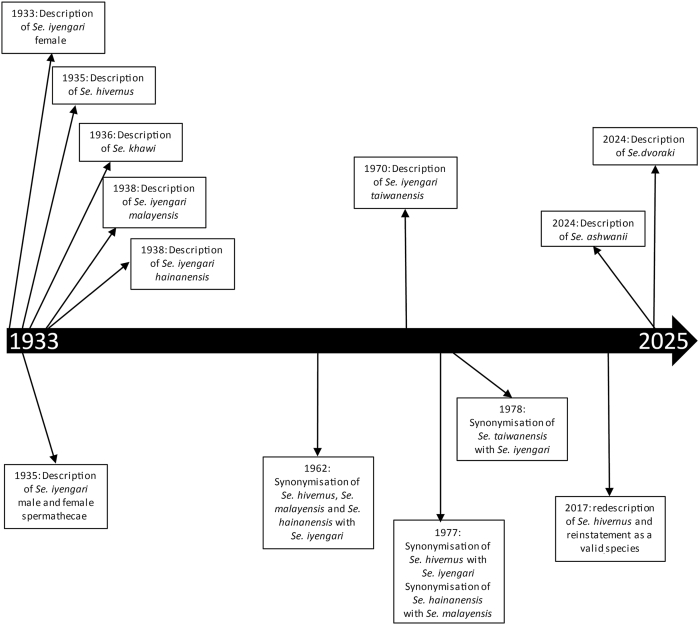
Historical timeline illustrating taxonomic changes within the *Se. iyengari* Group.

**Fig. 6:**
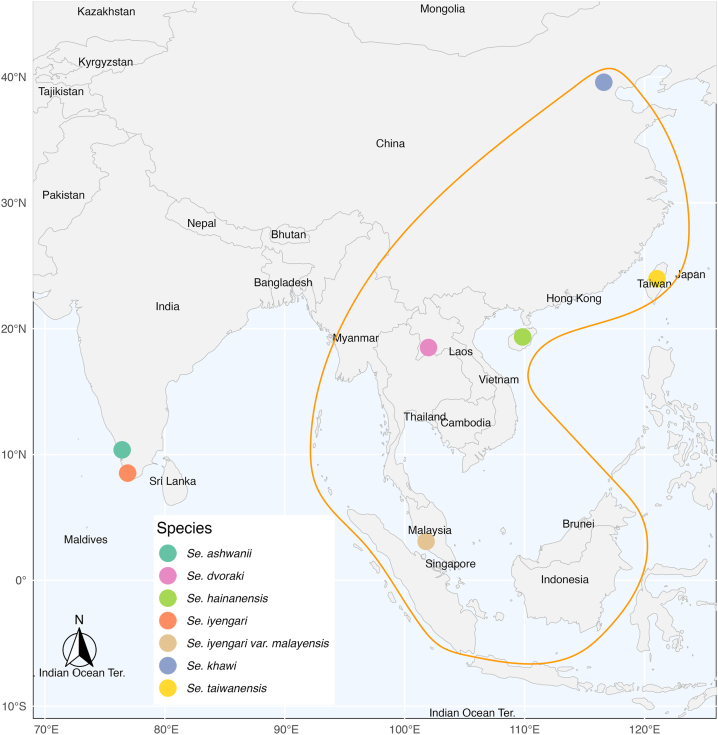
Limits of the distribution of the records of *Se. iyengari* (orange) and the position of the type-localities of the species *Se. iyengari* (*s.s*.), *Se. ashwanii*, *Se. dvoraki*, *Se. hainanensis*, *Se. iyengari malayensis*, *Se. khawi*, and *Se. taiwanensis*. *Sergentomyia hivernus* and *Se. gemmea* have been excluded from this group (Phumee et al., 2017; Depaquit et al., 2019).

The sandflies initially identified in Southeast Asia as belonging to the *Se. iyengari* Group differ in several key respects from Sinton’s original description (Sinton, 1933). The confused taxonomic history of this group, sometimes difficult to understand, is summarised in Fig. 5. Biogeographically, *Se. iyengari* was described from southern India, far from the Southeast Asian localities where similar specimens have been reported. Extensive entomological surveys in India have not confirmed its presence outside the type-region, making the occurrence of true *Se. iyengari* in Southeast Asia unlikely. Consequently, these biogeographical data lead us to believe that the species we found in Cambodia is not *Se. iyengari* (*s.s.*). We have positioned on a map (Fig. 6) the type-localities of the various species belonging to the *Se. iyengari* Group, including species whose synonymy with *Se. iyengari* and sometimes reinstatement have been proposed (Sinton, 1933; Raynal and Gaschen, 1935d, 1935c; Theodor, 1938; Yao and Wu, 1938; Quate, 1962; Cates and Lien, 1970; Leng, 1977; Lewis, 1978, 1987; Seccombe et al., 1993; Phumee et al., 2017; Depaquit et al., 2019; Saini et al., 2024).

From a morphological point of view, Sinton, in his description of *Se. iyengari* (Sinton, 1933), did not observe cibarial denticles, whereas they are systematically observed in samples from Cambodia, as well as on all the specimens from Southeast Asia. Moreover, in his original description, Sinton did not describe the spermathecae of this species. Raynal and Gaschen did this in 1935 (Raynal and Gaschen, 1935c), describing the spermathecae, as well as the male, based on samples from Vietnam. This description of the spermathecae in material coming from a location far removed from Sinton’s first mention leads to identification difficulties since the species described by Raynal and Gaschen (1935c) does not belong to *Se. iyengari* but probably to *Se. dvoraki*, a recently described species exhibiting spermathecae with a highly developed collar and the presence of a few vertical cibarial teeth (Vongphayloth et al., 2024). Recently, *Se. ashwanii* was described from southern India (Saini et al., 2024), very close to the type-locality of *Se. iyengari* (*s.s.*), without any discussion of its very close resemblance, or even synonymy with *Se. iyengari* (*s.s.*).

Among the samples examined in this study, cytochrome *b* sequences revealed two well-supported clades corresponding to geographically structured populations: *Se. iyengari* Group 1 and *Se. iyengari* Group 2. This difference at the molecular level distinguishes the samples captured in the south of the country (traps P1, P2 and P3 corresponding to *Se. iyengari* Group 2 population) from those captured in the middle of the country (traps C8 to C20 corresponding to *Se. iyengari* Group 1 population).

**Fig. 7:**
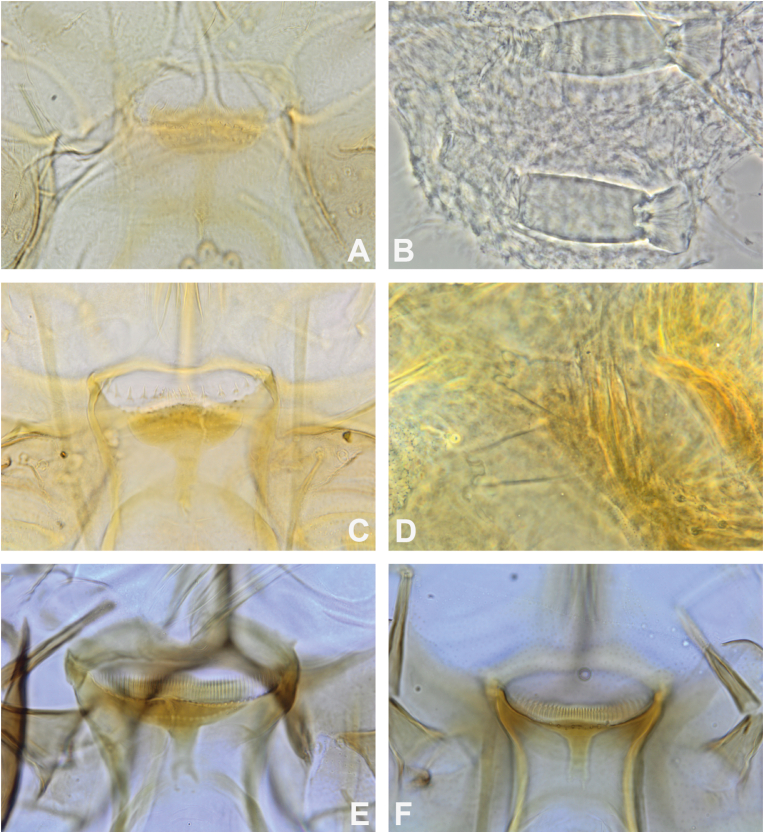
Cibarium (**A**, **C**, **D**, **E**) and spermathecae in females of *Se. iyengari* Group 2 labelled CAMB206 (**A**, **B**), *Se. iyengari* Group 1 labelled CAMB151 (**C**, **D**), and *Se. barraudi* Group 2 labelled CAMB233 (**E**), and *Se. barraudi* Group 1 labelled CAMB237 (**F**).

This assemblage is heterogeneous, and species-level assignment within the group remains uncertain. Over the years, its composition changed. Even today, the identification of these species is not straightforward. Morphologically, the differences in the spermathecae in females were observed, with the spermathecal head either being surrounded by a collar or not (Fig. 7B-D). In males, any characteristics differentiating the two molecular groups were not observed. Table 7 provides comparative data for the cibarium and spermathecae of the species belonging to the *Se. iyengari* Group.

**Table 7:** Comparison of the original descriptions of the cibarium and spermathecae in the species belonging to the *Se. iyengari* Group.

Species	Cibarium	Spermathecae
*Se. iyengari* (Sinton, 1933) (*s.s.*)	Well-developed sclerotised area. The main portion of this is shaped like a segment of an orange with the convexity forward. This convexity is continued forwards as a large blunt process, while from the middle of the straight posterior border arises a small, less highly chitinised, projection. The buccal armature has 4 small, narrow, central teeth and 5 large, broad ones on each side. These teeth are arranged in a row with the concavity backwards.	Not described in the original description, nor from Indian specimens.
*Se. khawi* (Raynal, 1936)	The sclerotised area is very distinct, shaped like a flattened German spiked helmet, with the point facing anteriorly. The row of teeth is sinuous, concave towards the posterior on the sides, then gradually becoming convex in its median part. There are about 20 fairly sharp teeth, 8–9 μm long and slightly separated from each other. Anteriorly, two or three rows of vertical teeth are visible.	Shaped like cylindrical bags with smooth walls, the spermathecae are three times longer than they are wide and have a cluster of setae at their tip, borne on an invaginated protuberance. Spermatheca length: 60 μm; maximum width: 20 μm. The individual ducts are wide (10 μm). Proximal end not observed.
*Se. iyengari malayensis* (Theodor, 1938)	A row of 15–17 long, pointed teeth arranged in an arc slightly concave posteriorly. The 4 median teeth are placed more closely together than the lateral ones. There is a second row of much smaller stumpy teeth anterior to the long teeth. The median ones of these small teeth are pointed and larger than the lateral ones. At the sides, there is sometimes a double or triple row of small teeth. In some cases, a third row of teeth seems to be present in the middle, corresponding in position to the median teeth of the second row. The sclerotised area stretches over the whole width of the buccal cavity, and it is rounded anteriorly with a moderately long, pointed anterior process.	Long cylindrical capsules with slightly crinkled surface, moderately thick walls, and a relatively long anterior process. In some specimens, there are traces of irregular internal segmentation.
*Se. iyengari hainanensis* (Yao & Wu, 1938)	Well-marked sclerotised area, the main part of which assumes a crescent shape with a long, pointed projection from its anterior convex surface. In the middle of the concave posterior surface of the sclerotised area, there is a small expansion, but it is not so well-developed as that shown in the specimens from India and Indo-China. The cibarial armature consists of a row of about 17 continuous teeth, which cannot be differentiated as small narrow central teeth and broad side teeth as in the case of *Se. iyengari.* Anterior to this row, there are 3–4 rows of vertical teeth.	Spermatheca with a very dilated head, invaginated into the body. Trumpet-shaped opening. No internal folds.
*Se. iyengari taiwanensis* (Cates & Lien, 1970)	Slender, about as wide at the arch as at the teeth, with a well-developed arch, armed with 15–20 strong, hook-like teeth with triangular bases; 2 rows of 11–16 vertical teeth and a third incomplete row of 5–7 vertical teeth. Sclerotised area almost rectangular, with 2 prominent, widely separated projections.	Difficult to observe in mounted specimens. Tubular, smooth and thin walls, with an obscure apical knob armed with a definite ring of fairly long hairs; when dissected it is tubular and “V”-shaped. A thin disc attached to the apex of the ring of hairs may be seen in stained specimens.
*Se. ashwanii* Saini, Shah, Jessu, Anns & Amju, 2024	Cibarium: ventral plate bears cibarial teeth (hind teeth) and denticles (fore-teeth); dorsal plate with light golden brown coloured pigment patch, which is funnel-shaped. No dorsal process. Cibarium bears 10–12 varying teeth. All teeth are arranged in a single row, tapering to fine points. There are 4–6 small denticles in the ventral plate, arranged in a row, adjacent to cibarial teeth.	Spermatheca with secretory cells at the distal end, narrow individual spermathecal duct, length undetermined and common spermathecal duct not evidently visible.
*Se. dvoraki* Randrianambinintsoa, Vongphayloth & Depaquit, 2024	Armed with 18 posterior teeth and two central rows of anterior teeth: 6 bigger and 6 smaller. Sclerotised area rounded, not reaching the lateral side of the cibarium. Sclerotised arc not observed.	Narrow wrinkled spermathecae, with thin walls. Terminal knob deeply embedded in the capsule. Short, smooth common duct. Long, smooth individual ducts.

Specimens assigned to “*Se. iyengari* Group 1” are morphologically similar to *Se. khawi*, particularly in spermathecal structure, which lacks a pronounced apical collar. However, the sclerotised area (formerly pigmented patch) of the female cibarium is in the shape of a well-marked crescent, with a long and thin anterior projection, which is not very visible. This does not correspond to the description of *Se. khawi* described as having the shape of a helmet with a flattened tip. Moreover, Cambodian specimens exhibit approximately 14 cibarial teeth, fewer than the 20 teeth reported by Raynal (1936) for *Se. khawi*.

Specimens assigned to “*Se. iyengari* Group 2” exhibit a well-developed and deep collar at the apex of the spermathecae, closely related to that described in *Se. hainanensis* (Yao and Wu, 1938). Indeed, Yao and Wu described a spermathecae with a very dilated head, invaginated into the body, and a trumpet-shaped opening, which we call the collar. The cibarial sclerotised area is well-marked, crescent-shaped, with a long, pointed area at its anterior part and a small extension in the middle of the posterior surface. The cibarial armature consists of approximately 17 teeth. Anteriorly, there are six small teeth and three to four rows of chitinous dots, which correspond to the denticles (Fig. 7A). In Cambodian specimens, both the spermathecae and the cibarium match this description. The deep collar around the apex of the spermathecae is clearly visible. In view of all these elements, caution is warranted regarding the identification of these species in the *Se*. *iyengari* Group, which deserves to be studied more precisely on a sample from multiple countries in Southeast Asia, even India. Indeed, taxonomic confusion has often arisen, with several species being associated with *Se. iyengari* initially described by Sinton (1933) despite numerous differences that could make them independent species. Therefore, the presence of *Se. iyengari* in Southeast Asia is considered unlikely. The molecular data (Fig. 2) confirm the strong differentiation of these two populations of *Se. iyengari* Group in Cambodia.

It would be interesting to focus on the study of this group in order to redefine the specific identification characteristics in light of the observation of the type-specimens. Despite significant efforts to locate the type-specimens of *Se. khawi* and/or the Raynal’s collection, no materials were found at the National Museum of Natural History in Paris, the Pasteur Institute in Paris, or in the National Institute of Hygiene and Epidemiology collections in Hanoi, Vietnam. It must therefore be concluded that these type-specimens either never existed, were never deposited, or have been lost. Therefore, resolving this taxonomic conundrum depends on the new collection, followed by morphological and molecular analysis of topotypes of *Se. khawi*, as well as all species in the *Se. iyengari* Group.

Molecular data confirm the differentiation of the two populations that could potentially be assigned a specific status: *Se. khawi* and *Se. hainanensis*. However, caution remains warranted regarding the identification of these species within the *Se. iyengari* Group. At present, further taxonomic speculation is avoided to prevent adding to existing confusion. Estimates of evolutionary divergence over sequence pairs between *Se. iyengari* Group 1 and *Se. iyengari* Group 2 is 0.029, raising the question about the potential species status of these two populations (Table 5).

*Sergentomyia hivernus* is a species whose previously suggested synonymy with *Se. iyengari* (Quate, 1962), which was both curious and awkward, has been shown to be erroneous, and its specific status is now clearly established (Phumee et al., 2017). While the cibarium and pharynx of the female closely resemble those of *Se. iyengari* (*s.l.*), the spermathecae are smooth and broad, with ducts that are of the same diameter throughout their length that converge into a common proximal canal. The head of the spermathecae is invaginated within a collar.

### 4.4 Genus *Sergentomyia*: Taxonomic confusion in the *Se. barraudi* Group

**Fig. 8:**
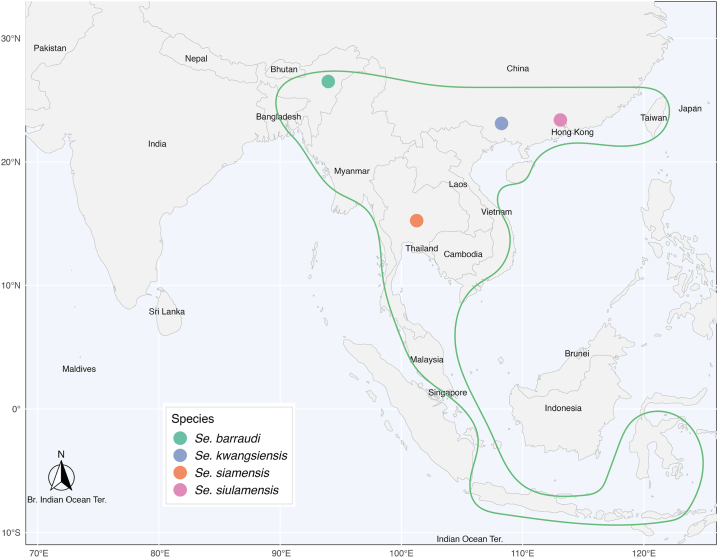
Limits of the distribution of the *Se. barraudi* Group (*green*) and position of the type-localities of the species *Se. barraudi* (*s.s.*), *Se. kwianguensis*, *Se. siamensis*, and *Se. siulamensis* constituting this group.

*Sergentomyia barraudi* is the main species of a group that bears its name, the *Se. barraudi* Group (Fig. 8). The species boundaries within this group remain questionable. In his original description of the female *Se. barraudi*, Sinton (1929) depicted a marked, mushroom-shaped pigmented area with a stem divided into two at its end. He counted about forty palisade-like cibarial teeth (Fig. 7E and F), organised on a line that is rather concave towards the rear (Sinton, 1929). The pharynx is only slightly dilated at its posterior part; it is bordered by numerous fine, long, and pointed teeth, forming a dense brush. The spermathecae are smooth.

The species *Se. siamensis* is very similar (Theodor, 1938). Causey (1938) described a pharyngeal armature in the female that is composed of slightly shorter teeth. In the cibarium, the teeth are also palisade-like, but in greater number, since there are 54 of them. The spermathecae are identical to those of *Se. barraudi*. Other species of the *Se. barraudi* Group were considered during the identification of the two observed groups: *Se. barraudi* var. *kwangsiensis* (Yao and Wu, 1941); and *Se. barraudi* var. *siulamensis* (Chen and Hsu, 1955). These species were later excluded due to the number of cibarial teeth, which is approximately 70 in each case.

**Fig. 9:**
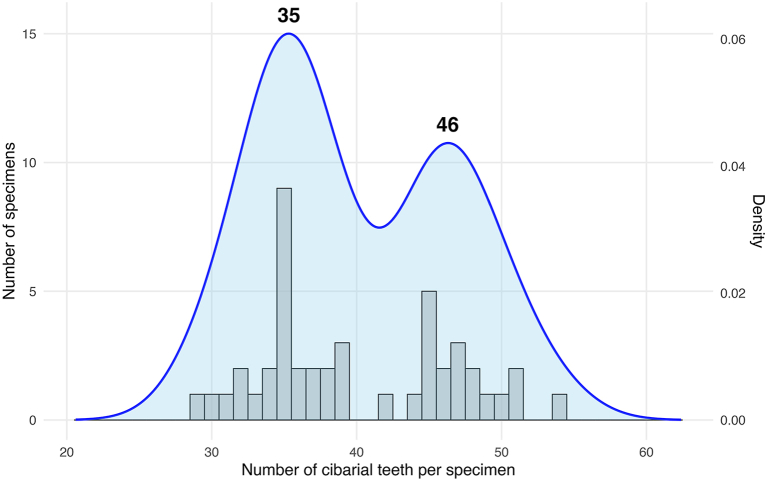
Distribution of the number of cibarial teeth of *Se. barraudi* and *Se. siamensis*. Bars represent the number of specimens found with the corresponding number of teeth. The blue line represents the density of the distribution. The bold numbers above each peak represent the number of teeth with a density peak. The graph was made with RStudio (*ggplot2* package) (Wickham, 2016; R Core Team, 2023).

In Cambodian samples, among females, two populations can be clearly distinguished by the number of teeth (Fig. 9) as well as by the molecular analysis (Fig. 2). The population called *Se. barraudi* Group 1 has fewer than 42 teeth, which should correspond to *Se. barraudi* (*s.s.*), while the individuals in the other population, called *Se. barraudi* Group 2, having more than 44 teeth should be related to the species *Se. siamensis*. However, this population of *Se. barraudi* Group 2 appear different from the *Se. siamensis* collected in Thailand and Laos (Fig. 2). In both species, the sclerotised area exhibits the anterior bifurcation.

The present study, based on cytochrome *b* sequencing, revealed six molecular populations within the *Se. barraudi* Group (Fig. 2), exceeding the number of species initially included in this group. Its wide distribution and the molecular variability observed in Southeast Asia raise questions about the taxonomic status of these molecular populations, as well as that of the four species previously included in this group. Based on the literature and previous observations, it appears that the taxa *Se. kwiangsiensis* and *Se. siulamensis* are not present in Southeast Asia, complicating the assignment of species names to these populations. To avoid further confusion, it seems reasonable to refer to these populations as the *Se. barraudi* Group, pending a comprehensive study involving the examination of type-specimens and molecular analysis of topotypes, which would allow a better understanding of the structure of this species complex, whose speciation processes are not yet complete.

### 4.5 Other *Sergentomyia* species

*Sergentomyia bailyi* is a species in which the female lacks a pigmented area or has a very small square spot (Fig. 3E). Numerous small, fine teeth are arranged in several rows, but this cibarial armature is sometimes difficult or even impossible to visualise, even using phase contrast at the highest magnification. The pharynx has a series of curved transverse ridges, with small, short teeth at their posterior margin. The anterior portion of the pharynx is narrow. The spermathecae are smooth, with a narrow duct.

The pharyngeal armature of the female *Se. sylvatica* consists of toothed, parallel folds on the sides, which then extend obliquely backwards and form an alveolar network in the centre. On the cibarium, a more or less rectangular, tall and narrow slightly sclerotised area is observed. The cibarial armature consists of about a dozen sharp teeth, larger in the middle than on the sides, and well-spaced from the other ones (Fig. 3F). The spermathecae are in the shape of cylindrical capsules. On the mouthparts, very well-developed teeth are observed on the maxillae and mandibles, somewhat resembling those of *Se. ingrami* (Abonnenc, 1972). This is a very pigmented species. The labial furca is closed and brown. The specimens observed in this study were collected from a cave, thus demonstrating the cave-dwelling nature of this species.

The female of *Se. anodontis* is described as having a thin and unarmed pharynx. The cibarium is also unarmed but is characterised by a median projection in the shape of an inverted “V” (Fig. 3C). The spermathecae consist of long tubules with no separation between the spermathecae and the duct. At the apex, a thick, hairy knob is invaginated. Below, several distal rings are visible. These characteristics make the female very, perhaps even too, easy to identify. Consequently, to our knowledge, no one has really taken a close look at this species. However, there is molecular variability (Fig. 2, Table 6) that suggests it is likely a species complex that deserves further study in light of a morphological and molecular analysis including several populations from its entire distribution range. The specimens from Cambodia are consistent with the original description, but a structure covering the inverted V-shaped median projection and the presence of small, translucent lateral teeth are observed.

### 4.6 Genus *Grassomyia*

Specimens of the genus *Grassomyia* are easily recognisable because they lack ascoids on the first flagellomere (= third antennal article), which is generally very short. Their wings are usually very narrow. They possess rounded, echinulate spermathecae, shaped like poppy capsules. Generally, these are well-pigmented species. Identification of a *Grassomyia* is easy at the generic level, but specific identification is often very difficult and sometimes requires the examination of thoracic characters. Never implicated in the transmission of *Leishmania* to humans, species of this genus have been the subject of a few studies.

A revision of the genus *Grassomyia* should be considered at a global scale, including populations from Africa, the Middle East and Southeast Asia. The species frequently reported in Southeast Asia is *Grassomyia indica* (Quate and Fairchild, 1961; Jariyapan et al., 2022; Preativatanyou et al., 2023; Sunantaraporn et al., 2024; Klaiklueng et al., 2025; Soomro et al., 2025). To prevent potential misidentification, and considering that specimens from Southeast Asia might be different from those from India, specimens from Cambodia are here referred to as *Grassomyia* sp. The species *Gr. indica* was originally described rather briefly as having 33 to 36 cibarial teeth (Theodor, 1931). Examination of 78 specimens from Cambodia revealed 19–50 teeth (mean: 31.2), raising questions about their taxonomic identity. However, these apparent morphological variations contradict the observed molecular homogeneity (Fig. 2). A large-scale morphological and molecular study of the genus *Grassomyia* would be valuable in the future.

This study provides the most comprehensive update on the diversity and distribution of phlebotomine sand flies in Cambodia. The detection of distinct morphological and molecular groups within both *Se. iyengari* and *Se. barraudi* suggests that multiple, potentially undescribed species occur in the region. To prevent future taxonomic confusion, specimens that cannot be confidently identified at the species level should be referred to as the *Se. iyengari* Group (or *Se. iyengari* (*s.l.*)) and the *Se. barraudi* group (or *Se. barraudi* (*s.l.*)). These findings underscore the complexity of sand fly species in Southeast Asia and highlight the importance of integrating morphological and molecular approaches for accurate species delimitation. Further studies, including examination of type-specimens and topotypic populations from multiple locations, are essential to resolve the taxonomy of these vector species.

## 5 Conclusions

The sand fly fauna of Southeast Asia remains poorly studied. Many misidentifications, attributed to closely related species, probably allopatric, appear to be common in the scientific literature. This region, quite different from India and northern China, constitutes, due to its geography (mountains isolating populations), a region highly conducive to vicariance speciation, a phenomenon that is very little studied in sand flies. This results in incomplete speciation, which largely explains the sand fly taxonomic complexity in this region. We draw attention to the need for great caution in the identification of sand flies and emphasize that it is sometimes preferable to indicate a group identification (i.e. *Se. iyengari* Group) rather than a more precise identification that might prove erroneous.

## Ethical approval

Not applicable.

## CRediT authorship contribution statement

**Didot Budi Prasetyo:** Investigation, Writing - review & editing. **Thibault Vallecillo:** Investigation, Methodology, Writing - review & editing. **Eva Krupa:** Investigation, Methodology, Writing - review & editing. **Julian Gratiaux:** Investigation, Methodology, Writing - review & editing. **Alicia Vol:** Investigation, Methodology, Writing - review & editing. **Mathieu Loyer:** Investigation, Methodology, Writing - review & editing. **Jean-Philippe Martinet:** Investigation, Methodology, Writing - review & editing. **Quentin Di Brasi:** Investigation, Methodology, Writing - review & editing. **Khamsing Vongphayloth:** Investigation, Methodology, Writing - review & editing. **Bruno Mathieu:** Investigation, Methodology, Writing - review & editing. **Sébastien Boyer:** Investigation, Methodology, Writing - review & editing. **Arezki Izri:** Investigation, Methodology, Writing - review & editing. **Frédérick Gay:** Investigation, Methodology, Writing - review & editing. **Jérôme Depaquit:** Conceptualization, Writing – review & editing, Supervision, Methodology, Project administration.

## Funding

This research did not receive any specific grant from funding agencies in the public, commercial, or not-for-profit sectors.

## Declaration of competing interests

The authors declare that they have no known competing financial interests or personal relationships that could have appeared to influence the work reported in this paper.

## Data Availability

All the sequences supporting the conclusions of this article have been deposited in GenBank under the accession numbers PX864485-PX864568.
